# An effective datasets describing antimicrobial peptide produced from *Pediococcus acidilactici* - purification and mode of action determined by molecular docking

**DOI:** 10.1016/j.dib.2020.105745

**Published:** 2020-05-22

**Authors:** Ramachandran Chelliah, Kandasamy Saravanakumar, Eric Banan-Mwine Daliri, Joong-Hark Kim, Jung-Kun Lee, Hyeon-yeong Jo, Inamul Hasan Madar, Se-Hun Kim, Sudha Rani Ramakrishnan, Momna Rubab, Kaliyan Barathikannan, Fred Kwame Ofosu, Hwang Subin, Park Eun-ji, Fazle Elahi, Myeong-Hyeon Wang, Deog-Hwan Oh

**Affiliations:** a Department of Food Science and Biotechnology, College of Agriculture and Life Sciences, Kangwon National University, Chuncheon, Gangwon-do 24341, Korea; b Department of Medical Biotechnology, College of Biomedical Sciences, Kangwon National University, Chuncheon, Gangwon-do 24341, Korea; c Erom, Co., Ltd., Chuncheon, Gangwon-do 24427, Korea; d School of Food Science and Biotechnology, Kyungpook National University, Daegu, South Korea; e Department of Biochemistry, School of Life Science, Bharathidasan, University, Thiruchirappalli, Tamilnadu, India

**Keywords:** antimicrobial peptide, mode of action, structural characterization, gene expression, primers designed based on protein motif, identification of antimicrobial peptide or functional peptide

## Abstract

Most of the probiotics Bacterial cells, express native antibacterial genes, resulting in the production of, antimicrobial peptides, which have various applications in biotechnology and drug development. But the identification of antibacterial peptide, structural characterization of antimicrobial peptide and prediction on mode of action. Regardless of the significance of protein manufacturing, three individual factors are required for the production method: gene expression, stabilization and specific peptide purification. Our protocol describes a straightforward technique of detecting and characterizing particular extracellular peptides and enhancing the antimicrobial peptide expression we optimized using low molecular weight peptides. This protocol can be used to improve peptide detection and expression. The following are the benefits of this method, (DOI – https://doi.org/10.1016/j.ijbiomac.2019.10.196 [1]).

The data briefly describe a simple method in detection identification, characterization of antimicrobial extracellular peptide, predicating the mode of action of peptide in targeting pathogens (*In-silico* method), brief method on profiling of antimicrobial peptide and its mode of action [1]. Further the protocol can be used to enhance the specific peptide expressions, detection of peptides. The advantages of this technique are presented below:

• Characterization protocol of specific antimicrobial peptide
• The folded antimicrobial peptide expression were less expressed or non-expressed peptides.
• Besides being low cost, less time-consuming, easy to handle, universal and fast to execute, the suggested technique can be used for multiple proteins expressed in probiotics (*Lactobacillus species*) expression system.

**Specifications table**

**Table:** 

**Subject**	Biotechnology, Microbiology, Molecular Biology
**Specific subject area**	The subject covers majorly on biotechnology, which includes – **Proteomics** - characterization of peptide, **molecular and microbiology field** - safety profiling of peptide.
**Type of data**	Table
	Image
	Chart
	Graph
	Figure
**How data were acquired**	● **Transmission electron microscope image (TEM)**
	Field emission TEM (JEM-2100F, JEOL) at the Korean Basic Science Institute, Chuncheon center; Samples were sectioned using and Ultra microtome (Ultra cut UCT, Leica)
	**● LC-ESI-TOF-MS/MS Mass spectrometry**
	(High-performance liquid chromatography - UltiMate 3000 Series, USA; an self-sampler (MDS SCIEX, Seoul, Korea); Integrated hybrid quadrupole-time-of-flight (TOF) mass spectrometer (Applied Biosystems, Seoul, Korea); Sample were entombed on a ZORBAX 300SB-C18 ruse column (5-µm particle size, 300-µm i.d × 5 mm, 100 pore size) Agilent Tech, USA; Capillary column (75-µm i. d × 150mm, 3.5µm subdivision size, 100 pore size, part number 5065-9911) (Zorbax 300SB-C18);
	**● Reversed-phase High-performance Liquid Chromatography (RP-HPLC)**
	Model - (Aglient 1260 series); Sample was applied to a Symmetry® C18 column (5 µm, 4.6 × 150mm, USA).
	**● UV-Spectrophometer**
	Quantification of protein -Coomassie Plus (Bradford) Assay Kit Coomassie Plus (Bradford) Assay Reagent (Product No. 23236), 950mL, containing coomassie G-250 dye, methanol, phosphoric acid and solubilizing agents in water; store at 4°C. Albumin Standard Ampules, 2mg/mL, 10 × 1mL ampules, containing Bovine Serum Albumin (BSA) at 2.0mg/mL in a solution of 0.9% saline and 0.05% sodium azide - Product No. 23209), measure the absorbance at 595nm
	**● Size exclusion chromatography**
	< 30Kda spin; < 10Kda spin; < 3Kda spin (Pall corporation Macrosep® Advance centrifugal device, USA)
	**Data were obtained through software**:
	**●** Protein model were spawned by SWISS-MODEL (http://swissmodel.expasy.org) are licensed under the **CC BY-SA 4.0** Creative Commons Attribution-Share Alike 4.0 International License.
	(SWISS-MODEL generates theoretical models by automated homology modelling (3D protein structures) techniques Computational Structural Biology Group at the SIB Swiss Institute of Bioinformatics at the Biozentrum, University of Basel)
	**●** The peptides were inspected using **QS 3.0 software** (Applied Biosystems, Seoul, Korea)
	**Data were obtained through Program**
	**●** 3D structure of peptide from **RCSB PDB:****5UKZ** and 3U1Y.
	**● Cluspro 2.0 web server** for protein-protein docking, psi Blast using Phyre2 software (protein sequence comparison)
**Data format**	Raw
	Analysed
	Filtered
**Parameters for data collection**	**●** Amino acid sequences similarity analysis.
	**●** LC-ESI-TOF-MS/MS (liquid chromatography-electrospray ionization ion-trap time-of-flight mass spectrometry) analysis of SPI-derived peptides.
	**●** Bacteriocins, the bold type indicates amino acids derived from the N&C-terminal part of the parental bacteriocin.
	**●** Quantification Peptide
	**●** The interaction analysis between Pediocin and LipoXc by molecular interaction
**Description of data collection**	**Experimental factor**: LC-ESI-TOF-MS/MS analysis of Bacterial-derived peptides (≤3 kDa) after *Pediococcus acidilactici* (*P. acidilactici*) fermentation; Amino acid sequences analysis through applying psi Blast using Phyre2; Standard protein concentration based on Bradford method; The protein-protein molecular interaction analysis between Pediocin with LipoXc (cell wall protein) and topoisomerase protein
	**Experimental features:** Pediocin structural characterization based on specific sequence in SWISS-MODEL (https://swissmodel.expasy.org), Class IIB bacteriocin - Amino acid sequences similarity of the isolated pediocin with pediocin PA-1, enterocin A, Enterocin SE-K4, Prebacteriocin SkgA2, and the Bacteriocin mundticin; LC-ESI-TOF-MS/MS analysis of Bacterial-derived peptides further sequence similarity were analysed based on psi BLAST(Basic Local Alignment Search Tool) (Phyr2); Protein concentration were estimated based on Bradford method; protein-protein interaction on Cross points and key residues analysis i). Model of bad angel; (ii). Model of c deviation; (iii). Model of outliers Ramachandra plot; (iv). Model of twisted non proline; (v). Model of rotamer outliers
**Data source location**	Kangwon National University, Gangwon-do, Chencheon, South Korea – 200701; 37.8813° N, 127.7300° E; Department of Food Science and Biotechnology, College of Agriculture and Life Sciences.
**Data accessibility**	All data are presented in this article and raw data were shared through file share.com
	https://figshare.com/s/a6bd0748a48581faf3ce**.**
	**Sup Table 1:** Class IIB bacteriocin - Amino acid sequences similarity of the isolated pediocin with pediocin PA-1, enterocin A, Enterocin SE-K4, Prebacteriocin SkgA2, and the Bacteriocin mundticin. For the hybrid bacteriocins, the bold type indicates amino acids derived from the N-terminal part of the parental bacteriocin, and the normal type indicates the amino acids derived from the C-terminal part of the parental bacteriocin.
	**Sup**Fig. 2: Selection of Lactic acid bacterial strain from human milk based on the antimicrobial activity and further screened and shortlisted for bioactive peptide analysis
	**Sup Fig. 3:** Disc-diffusion method to determine the antimicrobial activity based on zone of Inhibition
	**Sup Fig. 4:** Structure of the Pediocin determination based on the sequence
	**Sup**Fig. 5: Chromatogram - LC-ESI-TOF-MS Data
	**Sup Table 6:** Pediocin Peptide sequence profile - LC-ESI-TOF-MS Data.
	**Sup Table 7:** Pediocin Identities – repeated sequence similarity
	**Sup Table 8:** Antimicrobial peptide sequence similarity were compared using Phyre2 software
	**Sup Fig. 9:** Molecular docking Data - Pediocin peptide with gram negative cell wall protein
**Related research article**	Author's name - Ramachandran Chelliah
	Title - Unveiling the potentials of Bacteriocin (Pediocin L50) from Pediococcus acidilactici with Antagonist Spectrum in a *Caenorhabditis elegans* model.
	Journal- International Journal of Biological Macromolecules
	DOI – https://doi.org/10.1016/j.ijbiomac.2019.10.196

**Value of the Data**

• The data on the *P. acidilactici* (probiotic) fermentation is resourceful and can be utilized in understanding their potential biotechnological applications. Based on LC-ESI-TOF-MS/MS analysis use as probiotics.
• Based on the amino acid sequence derived by LCMS and HPLC, the biosynthesised secondary peptide based metabolite were identified and quantified.
• The data demonstrated here can be applied by other future researchers working or studying in the field of bioactive peptide identification, characterization and purification based analysis.
• The data presented expands the molecular interaction model of complex of bioactive peptide and cell wall of pathogens towards determining the antimicrobial mechanism of peptide

## 1 Data

**Bacterial progression condition**

The 2% of *Pediococcus* strain, were inoculated in De Man, Rogosa and Sharpe (MRS) broth and incubated at 37 ± 2°C for 24 h, further incubated samples were aseptically transferred into sterile round bottom centrifuge tubes and centrifuged at 4000g for 15 min and cell free supernatant (CFS) was collected and proceeded for purification of bioactive compound.

### 1.1 Purification of bioactive compound

**Fig. 1:**
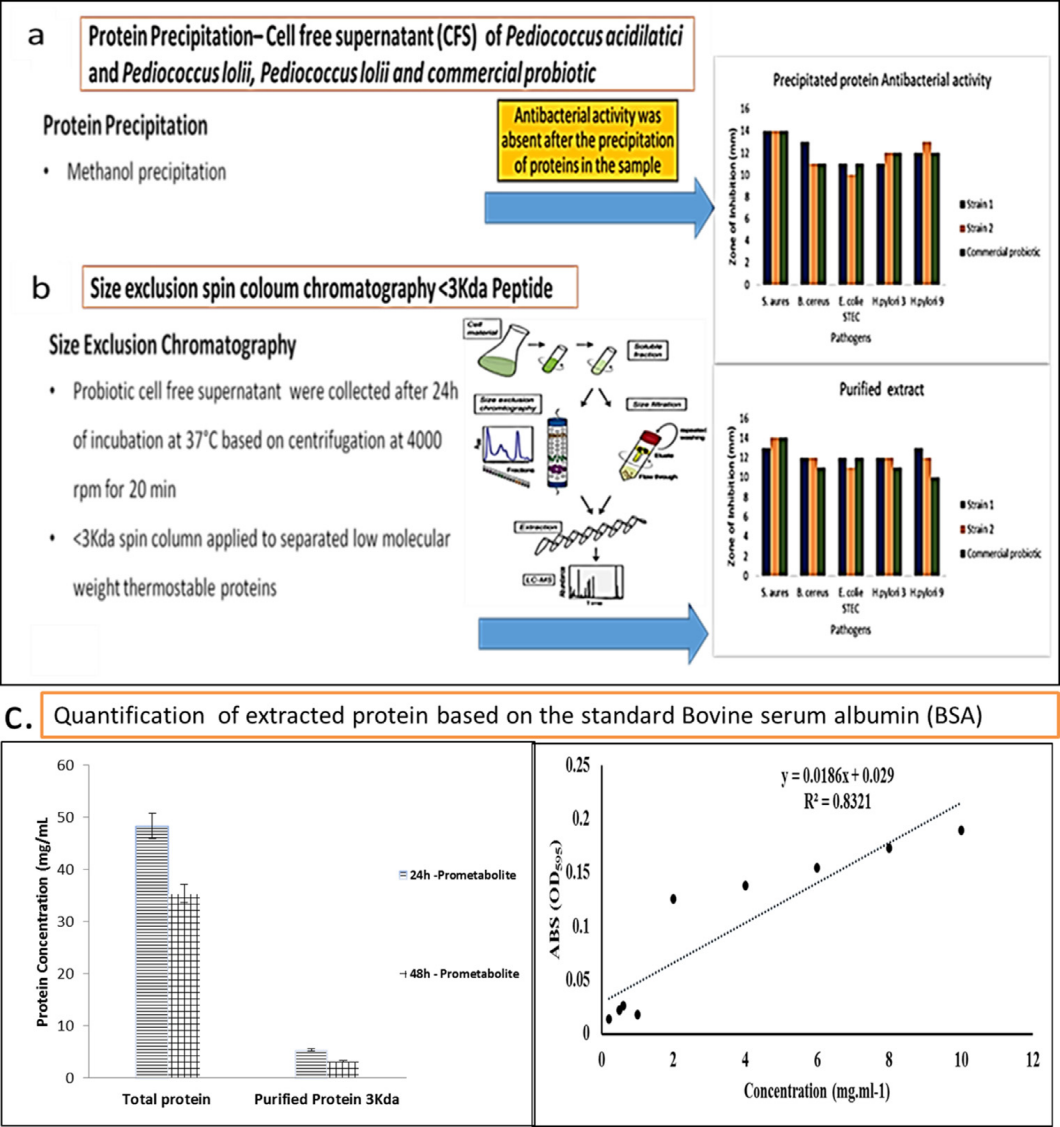
a The cell free supernatant (postbiotic) in which the proteins were precipitated based on addition of 70%methanol. 1b: Based on size-excluding spin column chromatography, a <30 Kda spin column with <3 Kda filtrate was subsequently applied to separate the low molecular weight (<3 Kda) antimicrobial peptides (AMPs). 1c: Quantification of extracted protein from 24 and 48h time interval based on the standard Bovine serum albumin (BSA).

**Fig. 2:**
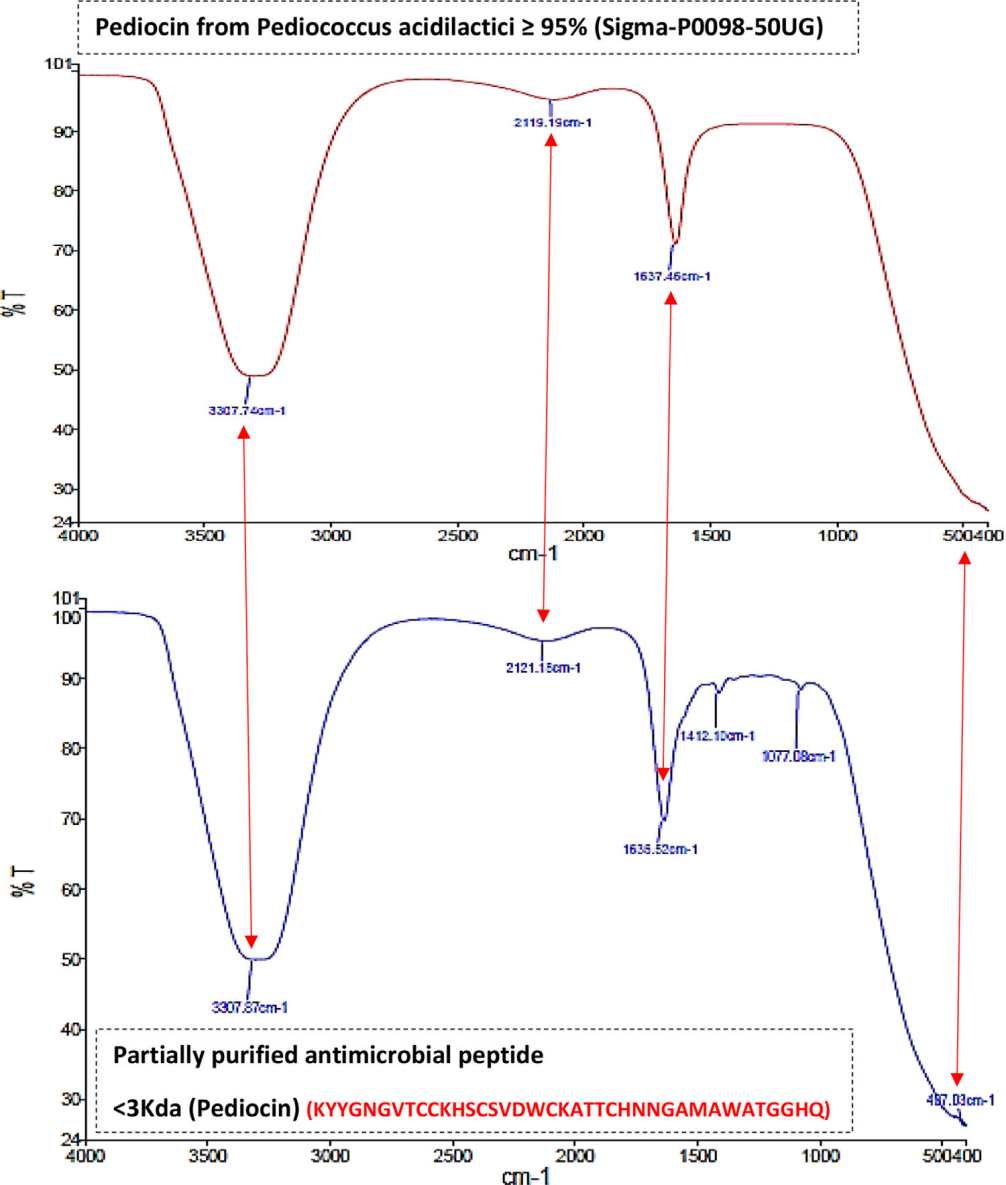
(a) FTIR of the Structural membrane permeabilizing Pediocin-Like Antimicrobial Peptide (AMP), (b) FTIR Structure of the membrane comparison of pediocin with <3Kda peptide purified from 24 h *Pediococcus acidilactici* strain.

The size exclusion spin column chromatography, <30Kda spin column subsequently with <3Kda filtrate was applied to separate the low molecular weight (<3Kda) antimicrobial peptide (AMP) was separated (Fig. 1), In addition the antimicrobial activity (Sup 2, 3)was analyzed using indicator foodborne pathogenic strains were analyzed and confirmed based on TEM analysis and exhibited inhibition efficiency comparatively higher with the 24h crude CSF extract and partially purified extract [Sup Fig. 6c copyright obtained from ramachandran et al. [1]. Further the functional group based structure were determined for extracted pediocin using Fourier-transform infrared spectroscopy (FTIR) (Fig. 2). The antimicrobial compound produced from *Pedicoccus*, Pediocin is sensitive bacterial cells and the mode of action shows bactericidal [Sup Fig. 6 copyright obtained from ramachandran et al. [1] activity. The molecular weight of pediocin falls under 2.7-17Kda, whereas pediocin PA-1 from *P. acidilactici* PAC1 was identified to be about 1.65Kda ^(^[2]^,^ [3]^)^.

### 1.2 Identification of peptides by mass spectrometry

**Fig. 3:**
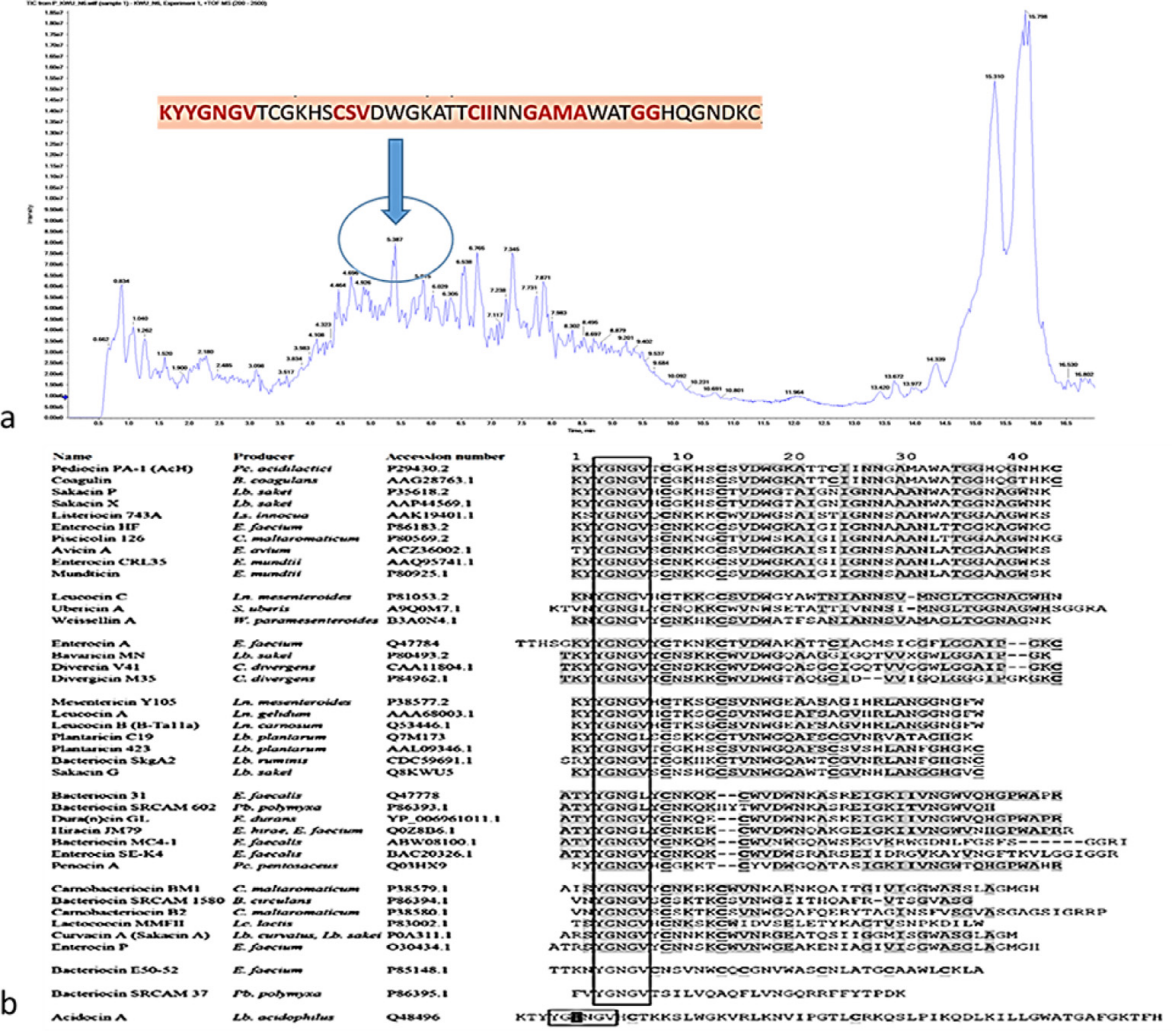
a LC-ESI-TOF-MS/MS of <3 Kda peptide purified - In total, 719 peptides were notorious and were eluted and tested in vitro for antimicrobial activity; the peptide sequences were screened using an in silico platform, pediocin protein and peptide profiling were developed in QS 3.0 software; 1b. Class IIB bacteriocin - Amino acid sequences similarity of the isolated Antimicrobial Peptide as Pediocin - Although many potential antimicrobial peptides were identified, the peptides KYYGNGV, FGNGV, NNGQV, ATGGGPVFGEE, and ATGGIPLELLTDKLKAL were the most abundant.

All the peptides were identified in the low molecular weight peptide profile and pediocin profiling were displayed in Table 1. Total 719 peptides were notorious and eluted, further tested in vitro for antimicrobial activity, the peptide sequences were screened using an in silico platform for pediocin protein and peptide profiling developed QS 3.0 software (Applied Biosystems, Seoul, Korea) to predict potential antimicrobial peptides (AMP). Although many potential antimicrobial inhibitory peptides were identified, peptides KYYGNGV, FGNGV, NNGQV, ATGGGPVFGEE and ATGGIPLELLTDKLKAL (Fig. 3) (Table1) (Sup 1, 4, 5, 6, 7) were most abundant. Which showed the strongest antimicrobial activity (0.268µg) of <3Kda fraction of CFS [Sup Fig. 6c copyright obtained from ramachandran et al. [1]. Which were not expressively different in their inhibitory abilities (p>0.05) when compared with commercial pediocin (sigma) [Sup Fig. 6 copyright obtained from ramachandran et al. [1]. Meanwhile, pediocin peptide (sigma - P0098-50UG) showed stronger inhibitory activity against *Helicobacter pylori* oki422 and *Escherichia coli* 0157 (STEC) [Sup Fig. 6 b, c copyright obtained from ramachandran et al. [1]. The antimicrobial (AM) mechanisms of peptide against foodborne pathogens [3]. Different mass-spectrometry peaks were identified to be encoded in *Lactobacillus* draft genome [4].

### 1.3 Stability of purified (pediocin) antimicrobial peptide and *In-vitro* simulated gastrointestinal digestion and analysis of digests by LC-ESI-TOF-MS/MS

**Fig. 4:**
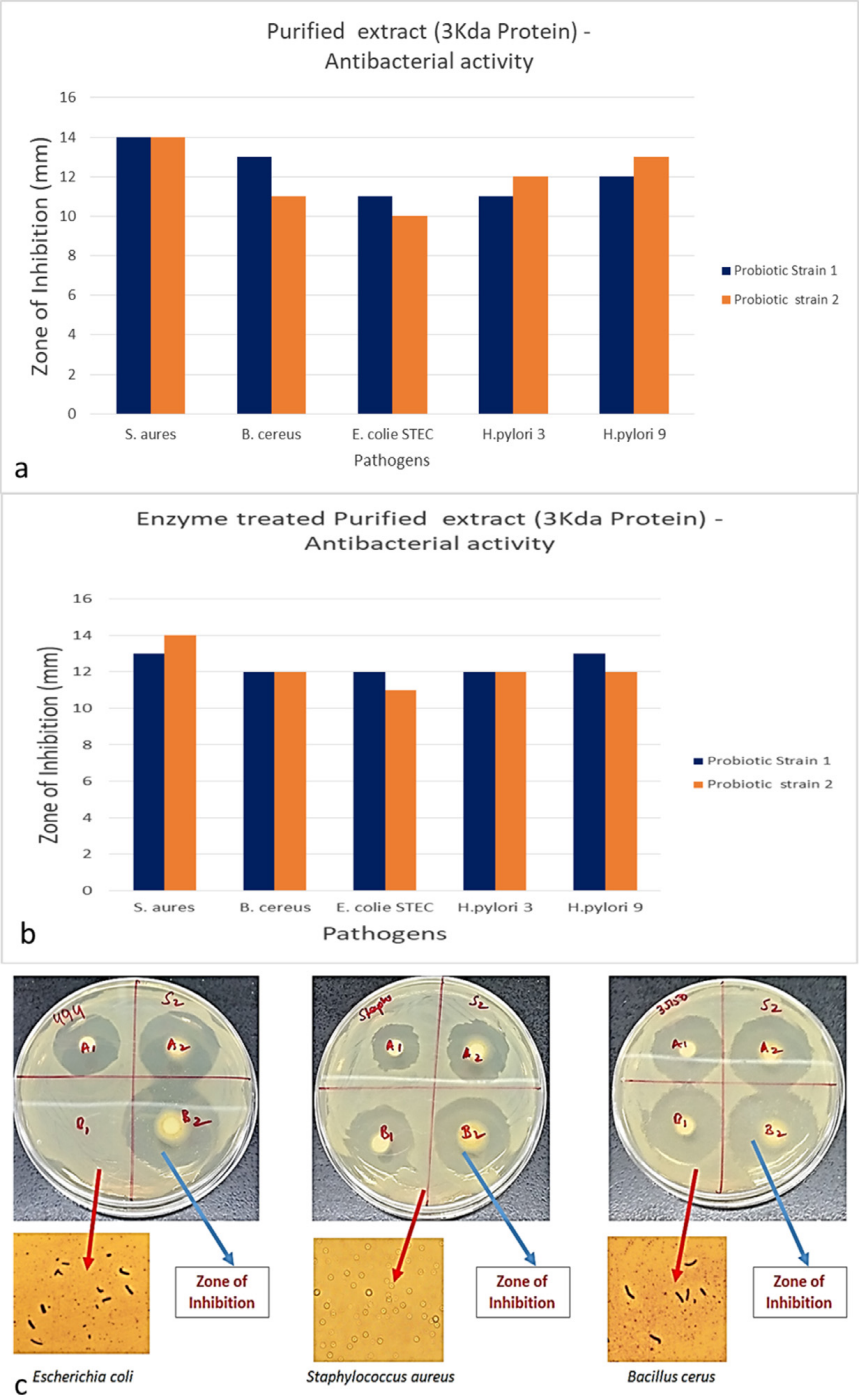
a: The low molecular weight (< 3Kda) fraction of cell free supernatant (CFS) purified based on spin column chromatography <3Kda; 4b. The low molecular weight (< 3Kda) fraction of CFS were subjected to pepsin digestion, its antimicrobial activity were not significantly changed; 4c. based on disc diffusion method the antimicrobial activity was determined for *Escherichia coli, Staphylococcus aureus, Bacillus cereus,* further the the zone of inhibiotion was observed under 100X phase contrast microscope.

The low molecular weight (< 3Kda) fraction of CFS were subjected to pepsin digestion, its antimicrobial activity will not be significantly changed. Also, successive treatment of digestive enzyme (peptides with pancreatin) did not affect the antagonist activity (p>0.05) as shown in (Fig. 4). The Mass spectrometry based on proteomics analysis and peptides were identified and categorized based on the sequence obtained and further the peptide, which survive in the digestive enzymes [5]. Potent antimicrobial defensing peptides are expressed in tissue-specific and constitutive and inducible ways in the Gastro intestinal condition [5] of humans.

### 1.4 Molecular interaction of antimicrobial peptide (AMP) towards Pathogens (Mode of Action)

**Swiss-model: homology modeling of protein structures**

The protein model were spawned by SWISS-MODEL are licensed under the CC BY-SA 4.0 Creative Commons Attribution-Share Alike 4.0 International License, based on the sequence generated by Liquid chromatography mass spectrometry (LCMS) SWISS-MODEL generates theoretical models [Sup Fig. 7a copyright obtained from ramachandran et al. [1].

**Fig. 5:**
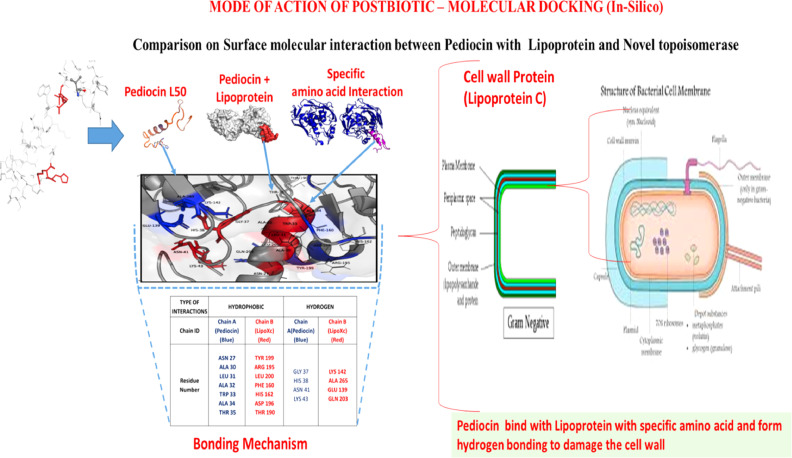
The three-dimensional (3D) structures of pediocin and LipoXc were downloaded from Protein Data Bank RCSB (Research Collaboratory for Structural Bioinformatics) (PDB: 5UKZ) (24) and 3U1Y. The Cluspro 2.0 web server for protein–protein docking, based on the fast Fourier transform correlation approach that evaluates docking confirmation by simple scoring functions. The N-terminal β-sheet, apparently due to a lack of unexplored regions, contains dual rigid hydrogen bonds among the hairpin β- loop and the 310 helix (His12-Gln39, 2.4 Å; Lys11-Gly40, 2.6 Å), which submits and interacts with the C-terminal chain to bend the peptide into a more rigid conformation.

The generated 3 dimensional structure of the pediocin analog 5 [Fig. 5b copyright obtained from ramachandran et al. [1] (Sup. 8, 9) is very similar to structures determined previously for other class IIa bacteriocins. It entails the N-terminal antiparallel β-sheet stabilized by the Cys9– Cys14 disulfide bond and a C-terminal tail folded into a α-helix [Sup. 5b copyright obtained from ramachandran et al. [1], which folds back onto the β-sheet to form a hairpin-like structure (Fig. 4 copyright obtained from ramachandran et al. [1]. The diverse class of antimicrobial peptides, specifically minor peptides were found to be rich in tryptophan (Trp) [6] were reported to be higher relative potency and specificity ^(^[7]^,^ [8]^)^.

## 2 Experimental design, materials, and methods

### 2.1 Purification of bioactive compound

The cell free supernatant (CSF) postbiotics from *Pediococcus* strain, incubated at 37 ± 2°C for 24 h was partially purified using Size exclusion chromatography (SEC). The CSF was filtered in the <30Kda spin column and centrifuged at 4000 rpm for 20 min. Further the <30Kda filtrate were transmitted to <3Kda tube (Pall corporation Macrosep® Advance centrifugal device, USA), and centrifuged at 4000 rpm for 15min one more [9]. Finally the filtrate was collected and tested for antimicrobial activity by applying disc diffusion method, through which partially purified low molecular weight peptide were tested against *Escherichia coli* 0157:H7, *Listeria monocytogenes* ATCC 15313, *Bacillus cereus* ATCC 14576, *Staphylococcus aureus* ATCC 19095 and *Helicobacter pylori* oki 422 and further the structure was confirmed based on the carbon Nuclear magnetic resonance (^13^C NMR).

### 2.2 Bradford protein assay

Materials and Reagents required for quantification of total protein as follows: Bovine Serum Abumin (BSA) (Sigma-Aldrich), coomassie Brilliant Blue G-250, methanol, phosphoric acid, bradford reagent (Dissolve 50 mg of Coomassie Brilliant Blue G-250 in 50 ml of methanol and add 100 ml 85% (w/v) phosphoric acid .Add the acid solution mixture slowly into 850 ml of H2O and let the dye dissolve completely, Store in a dark bottle at 4°C until use).

Standard assay procedure are as follows prepare five to eight dilutions of a protein (usually BSA) standard with a range of 5 to 100μg protein and dilute unknown protein samples to obtain 5-100μg protein/30μl. Further add 30μl each of standard solution or unknown protein sample to an appropriately labeled test tube. Set two blank tubes. For the standard curve, add 30μl water instead of the standard solution. For the unknown protein samples, add 30μl protein preparation buffers instead. Protein solutions are normally assay duplicate or triplicate. Add 1.5ml of Bradford reagent to each tube and mix were Incubated at room temperature (RT) for at least 5min. Absorbance will increase over time; samples showed incubate at RT for no more than 1h. Measure absorbance at 595nm.

Preparation of five standard solutions (1 ml each) containing 0, 10, 20, 30, 40 and 50μg ml BSA the pipette 800μl of each standard and sample solution (containing for <50μg ml protein) into a clean, dry tube. Protein solutions are normally assayed in duplicate or triplicate. Further add 200μl of dye reagent concentrate to each tube and vortex.

### 2.3 Precipitation and identification of secreted proteins

Precipitation of secreted proteins was achieved by adding minor modifications to the method described by Daliri et al [10]. Aliquots of 5mL of CFS and <3Kda peptide were harvested by centrifugation (10min, 3500g, 4^ᵒ^C), and the supernatant was filtered (0.45mm). Ten milligrams of sodium deoxycholate (Sigma- Aldrich Chimie, Saint-Quentin Fallavier, France) were added and mixed – the resulting solution was incubated at 4^ᵒ^C for 30min. Chilled TCA (Sigma-Aldrich Chimie) was added at a final concentration of 6% (w/v) and proteins were allowed to precipitate for 2h at 4°C. Proteins were recovered by centrifugation (9300g for 10min at 4°C) and pellets were washed twice with 2mL of chilled acetone (Sigma-Aldrich Chimie). Pellets were allowed to dry at room temperature (RT) and proteins were resolubilized by ultrasonication (10min, Ultrasonic bath, Deltasonic, South Korea).

### 2.4 Tricine SDS-PAGE Gel electrophoreses

Materials and Reagents required for the quantification of total protein as follows acrylamide, bisacrylamide, tris, tricine, Tetramethylethylenediamine (TEMED), ammonium persulphate, glycine, glycerol, urea, coomassie Brilliant Blue G-250 and protein Loading Buffer from south korea Spacer plates, short plates with comb(12well), Mini-PROTEAN (BioRad) Cell gel cassette, PowerPac Basic power supply and Polypeptide SDS-PAGE Standards (Bio-Rad)**,** Multicolored prestained low range protein ladder (Bio-Rad).

Composition for mini-gel was calculated referring to Tricine-SDS-PAGE protocol. To compare the effect of two glycerol concentration levels, Tricine-SDS gels of 10% and 13.3% glycerol(w/v), both of them with 18%, 3% separating gel, were casted, while 18%, 6% separating gels.

After optimizing the concentration of glycerol, five concentration levels of acrylamide-bisacrylamide (5%, 3%) were used to optimize the analytical electrophoresis condition. The separating gel was overlaid with 5%T, 3%C stacking gel.

Based on the Electrophoresis, the cathode buffer as the inner electrode buffer and anode buffer as the lateral electrode buffer. Polypeptide SDS-PAGE standards (Bio-Rad) comprised six proteins, covering the mass range of 1.4-23kDa. The concentration of polypeptide standards was about 8μg/μl. Total amount of 4μg proteins per well was applied for all Tricine-SDS gels, including the gels used to optimize the concentration of glycerol respectively.

Fixing, staining and distaining of Polypeptide bands were fixed in fixing solution for 30min (1.0-mm gels), then emerged in staining solution for 1h. An absolute background destaining of the 1.0-mm gels was performed by shaking the gels in 10% acetic acid for 2h. Destaining incubation lasted 30-60min with fresh de-staining solution and several times repeated. The fixing time of less than 45 minutes applied.

### 2.5 Tricine-Gel fraction and elution

Proteins were set in 5% (v / v) glutaraldehyde for 25 minutes. Three gel fractions between 1 and 15kDa have been cut into pieces of gel and proteolytic slurry. Lysate protein was precipitated overnight at −20°C and centrifugal at 14,000°C with acetone 1:5 (v / v) at 4°C, 10 minutes. Protein pellets (100μg) were resuspended into a buffer loading 25 μL (4% SDS, 20% glycerol, 10% 2-mercaptoethanol, 0.004% bromphenol blue and 0.125M Tris/HCl, pH 6.8) and incubated for 5min at 90°C.

### 2.6 Identification of peptides by mass spectrometry

Sequential identification of Peptides by LC-ESI-TOF-MS/MS (Mass Spectrometry Liquid chromatography-electrospray ionization-quantitative time-of-flight tandem mass spectrometry) were analyzed at the National Instrumentation Center for Environmental Management of Seoul National University in Korea, according to an earlier method Daliri et al [10]. Analysis were performed through applying HPLC (High-performance liquid chromatography- UltiMate3000 Series, USA), an combined arrangement encompassing an self-regulative nano pump, an self-sampler (MDS SCIEX, Seoul, Korea), integrated hybrid quadrupole-time-of-flight (TOF) mass spectrometer (Applied Biosystems, Seoul, Korea).The samples were ionized using nano-electrospray ionization. Further 1.5g of the P-SPI were dissociated in 50mL of sterile distilled water. Diverse elutes of fractions (1.5µL) of the sample were inserted in LC-nano ESI-MS/MS. The sample were entombed on a ZORBAX 300SB-C18 ruse column (5µm particle size, 300µm i.d × 5mm, 100 pore size) Agilent Tech, USA and washed for 6min graded with solvent-A [water/acetonitrile (98:2, v/v), 0.1% formic acid] 98% and solvent-B [Water/acetonitrile (2:98, v/v) 2% and 0.1% formic acid] at a flow rate of 5µL/min. The peptides were segregated on a capillary column (75-µm i. d × 150mm, 3.5µm subdivision size, 100 pore size, part number 5065-9911) (Zorbax 300SB-C18) at a transfer rate of 290nL/min with a gradient at 2%–35% solvent-B over 30 min, then from 35%–90% over 10min, followed by 90% solvent-B for 5min, and finally 5% solvent-B for 15min. The Electrospray were smeared by 2250eV based on coated silica tip. The peptides were inspected using QS 3.0 software (Applied Biosystems, Seoul, Korea). The ranges of proteins were identified based on 300–3000m/z values.

### 2.7 In vitro simulated gastrointestinal digestion and analysis of digests by LC-ESI-TOF-MS/MS

Double-stage simulated of enzyme digestion were performed based on Akash et al [11] with some modification. Mixture of both 200μg/1mL Pepsin with pH-2.0. The sample were incubated at 37°C for 2h and further the pH were adjusted to 7.5 with 0.2mg of Pancreatin were added to the sample were incubated at 37°C for 180 min. The reaction were terminated at 80°C for 10min and stored at −20°C till further analysis by LC-ESI-TOF-MS/MS as already described above (2.2)

### 2.8 Swiss-model: homology modelling of protein structures

The protein model were spawned by SWISS-MODEL are licensed under the CC BY-SA 4.0 Creative Commons Attribution-Share Alike 4.0 International License, based on the sequence generated by Liquid chromatography mass spectrometry (LC-ESI-TOF-MS/MS) [12] .

### 2.9 In silico – molecular interaction and docking

The 3D structure of Pediocin and LipoXc were downloaded from RCSB (PDB: 5UKZ [13] and 3U1Y [14]. Cluspro 2.0 web server for protein-protein docking based on Fast Fourier Transform correlation approach which evaluate docking confirmation by simple scoring functions [14] were applied for molecular docking simulations and predicting the biding affinity for Pediocin and LipoXc. Protein-protein docking based on the binding energy of surface properties, which in turn gives the protein interface probability and possibly gives the interaction site of two docked protein complex. Surface Racer 5.0 software calculates exacts Accessible Surface Area (ASA), Molecular Surface Area (MSA), cavities to the inner protein inaccessible to solvent from outside [15]. From the larger cluster weighted results from cluspro, top 5 ranked of clusters are taken from cluspro and run in the surface racer using S option‘s’ denotes van der waal's radii and we took‘s=2’ based on number of protein contained in ‘Complex_structure.pbd’ docked model had given, ‘r’ denotes radius and is 1.4 for all models, ‘m’ taken as 3 for all models. From the output MSA was taken and found best matching model using the following formula: [MSA (ligand) + MSA (receptor)-MSA (docked model)]. The formula were applied for larger surface area model is taken for further calculation [15]. The Ligplot+ version 2.1 [15,1] software was used under academic user license, the DIMPLOT program of LigPlot+ GUI was executed for obtaining plots of interactions across a selected protein-protein or domain-domain interface.

## Declaration of Competing Interest

There is no conflict of interest between the authors.
